# Evaluation of the Immunogenicity of a Pool of Recombinant *Lactococcus lactis* Expressing Eight Antigens of African Swine Fever Virus in a Mouse Model

**DOI:** 10.3390/vetsci12020140

**Published:** 2025-02-07

**Authors:** Jingshan Huang, Tianqi Gao, Zhanhao Lu, Dailang Zhong, Mingzhi Li, Hua-Ji Qiu, Yongfeng Li, Hongxia Wu, Yuan Sun

**Affiliations:** 1 State Key Laboratory for Animal Disease Control and Prevention, National African Swine Fever Para-Reference Laboratory, Harbin Veterinary Research Institute, Chinese Academy of Agricultural Sciences, Harbin 150069, China; hjs45333@163.com (J.H.); 19990736668@163.com (T.G.); luzhanhao106@163.com (Z.L.); hsrzlxcrb2021zdl@163.com (D.Z.); lmz824925@163.com (M.L.); qiuhuaji@caas.cn (H.-J.Q.); liyongfeng@caas.cn (Y.L.); 2 College of Animal Science and Technology, Jilin Provincial Engineering Research Center of Animal Probiotics, Key Laboratory of Animal Production and Product Quality Safety of Ministry of Education, Jilin Agricultural University, Changchun 130118, China

**Keywords:** African swine fever virus, *Lactococcus lactis*, immunogenicity, mouse model

## Abstract

African swine fever (ASF), caused by African swine fever virus (ASFV), poses a great threat to the global pig industry. There is an urgent demand for effective and safe vaccines to address this threat. This study reports the generation and evaluation of a recombinant *Lactococcus lactis* pool, each strain expressing one of eight ASFV antigens (F317L, H171R, D117L, E120R, B602L, CD2v, p54, and p72). We evaluated the immune responses in mice through oral gavage and intramuscular immunization to the recombinant *L. lactis* pool. The results show that the mice immunized via intramuscular injection induced high-level serum IgG antibodies within 7 d post-primary immunization, which was maintained over an extended period. Additionally, there was a marked increase in the interferon gamma (IFN-*γ*) and interleukin 10 (IL-10) levels in the sera. In contrast, the mice immunized via oral gavage did not induce obvious serum IgG antibodies. However, they experienced a transient peak of secretory IgA (sIgA) antibodies in fecal samples within 7 d post-primary immunization, which subsequently decreased to levels that were statistically similar to those in the control group. In addition, this study also found that the multi-antigen cocktail vaccination was safe for mice. This study provides a reference for the development and immunization of ASF vaccines with *L. lactis* as live carriers.

## Simple Summary

In light of the urgent need to combat the global threat posed by African swine fever (ASF) to the pig industry, this study generated a pool of recombinant *Lactococcus lactis* expressing eight distinct proteins of African swine fever virus (ASFV), the causative agent of ASF, and evaluated its immunogenicity in mice via both oral gavage and intramuscular injection. The results demonstrate that intramuscular immunization induced high levels of serum IgG antibodies in the mice, while oral immunization resulted in a transient increase in secretory IgA (sIgA) antibodies in mouse feces.

## 1. Introduction

African swine fever (ASF), caused by African swine fever virus (ASFV), is a highly contagious and hemorrhagic disease. It exhibits a diverse spectrum of clinical manifestations, ranging from chronic to peracute forms, and poses a significant threat to the stability and development of the global swine industry stability [1,2]. To mitigate ASF-associated losses, the development of safe and efficacious vaccines is imperative. The current landscape of ASF vaccine development encompasses various strategies, such as inactivated vaccines, live-attenuated vaccines, subunit vaccines, and live vector vaccines [3,4,5,6,7]. However, these vaccine strategies still face some limitations that necessitate addressing. For instance, while live-attenuated ASF vaccines are perceived to exhibit better efficacy, they entail higher safety risks due to potential residual virulence [8,9]. Conversely, subunit ASF vaccines have higher safety, but their protective efficacy still requires improvement [10,11]. Therefore, there is a need for continued research and development to address these challenges and ultimately produce a safe and effective ASF vaccine.

ASFV is a double-stranded DNA virus and the sole known tick-borne DNA virus [12]. The genome of ASFV spans a size range of 170 to 194 kilobase pairs (kb) and contains between 151 and 167 open reading frames (ORFs) [13]. ASFV encodes over 60 structural proteins and 100 non-structural proteins, which provide significant assistance in its assembly, replication, repair, and immune evasion [14]. Although the functions of some of these proteins remain unclear, numerous immunogenic candidate protective antigens have been identified. Among them, the ASFV p72 (encoded by the *B646L* gene), p54 (encoded by the *E183L* gene), p30 (encoded by the *CP204L* gene), and CD2v (encoded by the *EP402R* gene) proteins are the most commonly used protective antigen targets for ASF [15,16,17]. Furthermore, researchers systematically evaluated the immune responses of pigs to individual ASFV antigens by priming with a DNA vaccine via delivery of individual genes, followed by booster immunization using a recombinant vaccinia virus vector. They conducted an exhaustive screening of approximately 30% of ASFV antigens. The results indicated that the D117L and E120R proteins have potential as significant inducers of protective antibody responses or as serological markers of infection. Additionally, the F317L, B602L, p72, p54, and CD2v proteins were found to effectively stimulate specific lymphocytes in immunized pigs, eliciting a robust cellular immune response [18].

*Lactococcus lactis*, owing to its safety and ability to induce mucosal immune responses, has been widely employed in the development of mucosal vaccines. *L. lactis*, a well-studied lactic acid bacteria (LAB), holds the generally recognized as safe (GRAS) status due to its extensive history of use in human food fermentations and products [19]. This makes *L. lactis* widely used, including as a carrier for mucosal vaccines to present bacterial and viral antigens [20]. The nisin-controlled gene expression (NICE) system, a tightly regulated inducible expression platform, enables precise heterologous protein production in *L. lactis*, facilitating its genetic engineering for vaccinology [21]. Numerous studies have demonstrated the potential of *L. lactis* as a vaccine vector. For instance, recombinant *L. lactis* (r*L. lactis*) expressing multi-epitope antigens of foot-and-mouth disease virus (FMDV) serotype A was found to effectively induce the production of secretory IgA (sIgA) and IgG in mucosal tissues while also eliciting robust cellular immune responses [22]. In addition, *L. lactis* activates innate immune pathways, as evidenced by strain *L. lactis*-pNZ8148-BLS-Usp45, which expresses the *Brucella* LS protein, induced IgG antibodies and high levels of cytokines, such as interferon gamma (IFN-*γ*), tumor necrosis factor alpha (TNF-*α*), interleukin 4 (IL-4), and IL-10 [23]. Another study showed that using *L. lactis* as an oral vaccine delivery vector to express the VP6 protein of grass carp reovirus type II resulted in significant increases in the survival rate (46.7%) and protection rate (42.9%) compared with the control groups [24]. Researchers have also constructed r*L. lactis* strains capable of expressing *Eimeria tenella* immune mapped protein-1 (EtIMP1), a protective antigen against coccidiosis in chickens. Animal trials showed that compared to *L. lactis* carrying the parental vector, all three strains expressing EtIMP1 significantly reduced oocyst shedding, mitigated pathological changes in the cecum, and improved weight gain [25].

In this study, we selected eight immunogenic proteins of ASFV (F317L, H171R, D117L, E120R, B602L, CD2v, p54, and p72) to construct ASF vaccine candidates using *L. lactis* as a vector, and then evaluated the immune responses of these vaccine candidates in mice through both oral gavage and intramuscular injection routes. The objective was to establish a foundation for developing an oral vaccine using *L. lactis* as a carrier. By exploring the immune response capabilities induced by these r*L. lactis* strains, we aim to present new ideas and directions for advancing the development of an ASF vaccine.

## 2. Materials and Methods

### 2.1. Bacterial Strains, Plasmids, and Target DNAs

The nisin-inducible expression plasmid pNZ8149 and the *L. lactis* NZ3900 strain used in this study were generously provided by Dr. Hongyu Cui at the Harbin Veterinary Research Institute (HVRI) of the Chinese Academy of Agricultural Sciences (CAAS). The pNZ8149 plasmid is non-resistant, and the recombinant bacterial strains harboring pNZ8149 plasmid were screened by using a lactose-containing medium. The gene sequence of the ASFV HLJ/18 strain (accession number: MK333180.1) was retrieved from the GenBank database and optimized based on the whole genome sequence of the *L. lactis* MG1363 strain (the parental strain of *L. lactis* NZ3900). The optimized ASFV gene sequences were then synthesized by Nanjing GenScript.

### 2.2. Construction of the Recombinant Plasmids

The primers listed in Table 1 were used to amplify the linearized vector fragments from pNZ8149 and the target genes for ASFV. The resulting DNA fragments were separated by agarose gel electrophoresis and purified using a gel extraction kit (Omega, Norcross, GA, USA). The purified fragments were then subjected to homologous recombination using the ClonExpress II One Step cloning kit (Vazyme Biotech Co., Nanjing, China) to construct the recombinant plasmid [26].

### 2.3. Construction of the Recombinant L. lactis

The electrocompetent cells of the *L. lactis* NZ3900 strain were prepared following the method described previously [27]. Subsequently, *L. lactis* electrocompetent cells were chilling on ice until they were fully thawed. A total of 10 μL of the recombinant plasmid DNA was gently mixed with 50 μL of electrocompetent cells and incubated on ice for 2 min. Thereafter, the mixture was transferred into a pre-cooled 0.2-cm electroporation cuvette. The electroporation settings were adjusted to deliver a single pulse of 2000 V, 25 μF, and 200 Ω, with a single electrical pulse duration of 4 to 5 ms. Post-electroporation, the cells were promptly resuspended in 700 μL of pre-cooled GM17 broth (supplemented with 20 mM of MgCl_2_ and 2 mM of CaCl_2_), and placed on ice for 5 min. Activation ensued in a 30°C incubator over a period of 1 h. Following this, the culture was centrifuged at 6000*× g* for 2 min and resuspended in 50 μL of fresh GM17 medium. The resuspended cells were plated onto lactose-supplemented EM agar medium and statically incubated in a 30°C incubator for 24 to 48 h. Subsequently, single colonies were streaked onto fresh selective plates for purity verification prior to overnight culture in GM17 at 30°C. The plasmids were extracted for gene sequencing, and the r*L. lactis* strains with correctly matched sequences were preserved at −80°C for future use.

### 2.4. Western Blotting

To determine the subcellular localization of the recombinant proteins expression, the r*L. lactis* strains were induced with nisin to express the ASFV proteins, lysed by ultrasonication, and then fractionated into soluble (supernatant) and insoluble (precipitate) fractions by centrifugation. Equal volumes of 40 µL of the supernatant and precipitate were mixed with 10 µL of 5× sodium dodecyl sulfate-polyacrylamide gel electrophoresis (SDS-PAGE) loading buffer and boiled for 10 min. The samples were then subjected to SDS-PAGE separation and subsequently transferred onto a nitrocellulose membrane. The membrane was incubated with mouse anti-Strep antibodies (1:2000) as the primary antibody, followed by a goat anti-mouse IgG antibody (1:10,000) (Abcam, Cambridge, UK) as the secondary antibody. The blots were subsequently scanned using the Odyssey scanning imaging system.

### 2.5. Growth Curve of rL. lactis Strains

In order to evaluate the growth characteristics of the eight r*L. lactis* strains, we selected r*L. lactis*-F317L as a model organism. Overnight cultures of r*L. lactis*-F317L, *L. lactis*-pNZ8149 (strain containing an empty vector plasmid), and *L. lactis* NZ3900 (plasmid-free strain) were inoculated into EM broth at a 1:20 dilution ratio. Both induced and non-induced groups were established. The optical density at 600 nm (OD_600nm_) values of the cultures were measured initially, and subsequently recorded at hourly intervals until the bacterial stationary phase was reached. Utilizing these data points, growth curves were drawn for the r*L. lactis*-F317L, *L. lactis*-pNZ8149, and *L. lactis* NZ3900 under both the induced and non-induced conditions.

### 2.6. Immunization of Mice

A total of 20 specific-pathogen-free (SPF) six-week-old BALB/c mice were randomly divided into 4 groups, with 5 mice per group, as follows: (A) in the r*L. lactis* oral gavage group, each mouse was oral gavage-immunized with 8 × 10^9^ colony forming units (CFU) of a mixture of r*L. lactis*-F317L, r*L. lactis*-H171R, r*L. lactis*-D117L, r*L. lactis*-E120R, r*L. lactis*-B602L, r*L. lactis*-CD2v, r*L. lactis*-p54, and r*L. lactis*-p72 at a dose of 10^9^ CFU per strain; this mixture was termed the r*L. lactis*-mix; (B) in the r*L. lactis* intramuscular group, the immunogen and immunization doses were the same as in group A, but immunization was carried out through intramuscular injection; (C) in the *L. lactis*-pNZ8149 group, each mouse was oral gavage-immunized with 8 × 10^9^ CFU of *L. lactis*-pNZ8149; (D) in the PBS group, each mouse oral gavage-immunized with 200 μL of PBS. Among them, the r*L. lactis* intramuscular group was intramuscularly injected after the strains were inactivated, and the other groups of strains were immunized with live bacteria via oral gavage immunization. The immunization schedules are shown in Table 2.

The immunization schedule for the mice comprised the following steps: primary immunization, followed by oral gavage immunization on the subsequent day for a continuous period of 14 d. Two weeks later, a booster immunization was administered via oral gavage for three consecutive days. After another two weeks, a second booster immunization was given for three days. Each immunization was accompanied by a single intramuscular injection. Blood samples were collected from the orbital venous plexus on Day 0, Day 21 (7 d post-primary immunization), Day 37 (7 d post-first booster immunization), Day 44 (14 d post-first booster immunization), and Day 54 (7 days post-second booster immunization). On Day 54, the mice were anesthetized with CO_2_ and euthanized by cervical dislocation. Their spleens were harvested for subsequent lymphocyte proliferation assays.

### 2.7. Enzyme-Linked Immunosorbent Assay (ELISA)

Blood and feces samples of the mice were collected at various time points post-immunization. The collected blood samples were placed in a 37°C incubator for 2 h and centrifuged at 1000*× g* for 15 min. The supernatant obtained, which is the serum, was collected for the next detection. Regarding the feces samples, they were first weighed, and 0.4 mL of feces extract (prepared with 0.05 M EDTA-Na2 solution in PBS) was added to each 0.1 g feces. After shaking for 10 min, it was placed overnight at 4°C, centrifuged at 12,000*× g* for 5 min, and the supernatant was collected for the next detection. We used the prokaryotic expression system to express and purify the eight ASFV proteins in this study, with *Escherichia coli* Rosetta as the expression host and the pCold-TF plasmid as the expression plasmid.

The 96-well microplates were coated with the purified ASFV protein and incubated at 4°C overnight. Then, each well was washed three times with 200 μL of PBS containing 0.05% tween 20 (PBST). After washing, 150 μL of blocking solution (5% skimmed milk) was added to each well and incubated in a 37°C incubator for 2 h. The blocking solution was then discarded, and the microplates were dried. Next, 100 μL of the treated serum samples or fecal supernatants were added to the microplates (using reference anti-ASFV sera as the positive control and SPF pig sera as the negative control), and they were incubated in a 37°C incubator for 1 h. After incubation, the microplates were washed five times with PBST, 3 min each time, and then dried. Subsequently, 100 μL of HRP-conjugated rabbit anti-mouse IgG or goat anti-mouse IgA (Abcam, Cambridge, UK) was added into each well (the control sera were used with goat anti-pig IgG), and the microplates were incubated in a 37°C incubator for 45 min. After incubation, the microplates were washed five times with PBST for 3 min each time. The tetramethylbenzidine (TMB) chromogenic solution was added, 100 μL per well, and the microplates were kept at room temperature in the dark for 15 min. The reaction was stopped by adding 50 μL of 2 M H_2_SO_4_ to each well [28]. The concentrations of cytokines IL-2, IL-4, IL-10, and IFN-*γ* in the sera were quantified using a commercially available ELISA kit (Cloud-Clone, Wuhan, China).

### 2.8. Spleen Lymphocytes Proliferation Test

Spleen lymphocytes separation medium (TBD, Tianjin, China) was used to isolate the spleen lymphocytes of the mice after euthanasia. In brief, the splenic lymphocytes were suspended in RPMI-1640 medium supplemented with 10% fetal bovine serum (FBS) and 5% penicillin/streptomycin (Gibco, Waltham, MA, USA). The cells were then cultured in 96-well microplates at a density of 5 × 10^6^ cells per mL with 50 μL/well. The splenic lymphocytes were stimulated with 10^5^ TCID_50_ ASFV as a specific antigen stimulation, concanavalin A (ConA) (10 μg/mL) as a positive control, and RPMI-1640 medium as a negative control, respectively. The plates were then incubated for 72 h in a 5% CO_2_ incubator at 37°C. To assess the proliferation responses of the splenic lymphocytes, the cell counting kit-8 (CCK-8) assay (Ape×Bio, Houston, TX, USA) was used, after incubation at 37°C in an incubator in the dark for 3 h, the absorbance at 450 nm was read on a microplate reader. The data were presented as stimulation indices (SI), calculated as the ratio of the (stimulated value − blank value)/(unstimulated value − blank value).

### 2.9. Statistical Analysis

The results are presented as the means ± standard deviations (SDs). Statistical evaluations were conducted using GraphPad Prism version 9. To discern statistically significant variations among the various groups, a one-way analysis of variance (ANOVA) was employed, and in the text, ns denotes *p* ≥ 0.05, indicating no significance, * denotes *p* < 0.05, ** denotes *p* < 0.01, *** denotes *p* < 0.001, and **** denotes *p* < 0.0001.

## 3. Results

### 3.1. Eight rL. lactis Strains Enabled Soluble Expression of Corresponding ASFV Antigens

**Figure 1:**
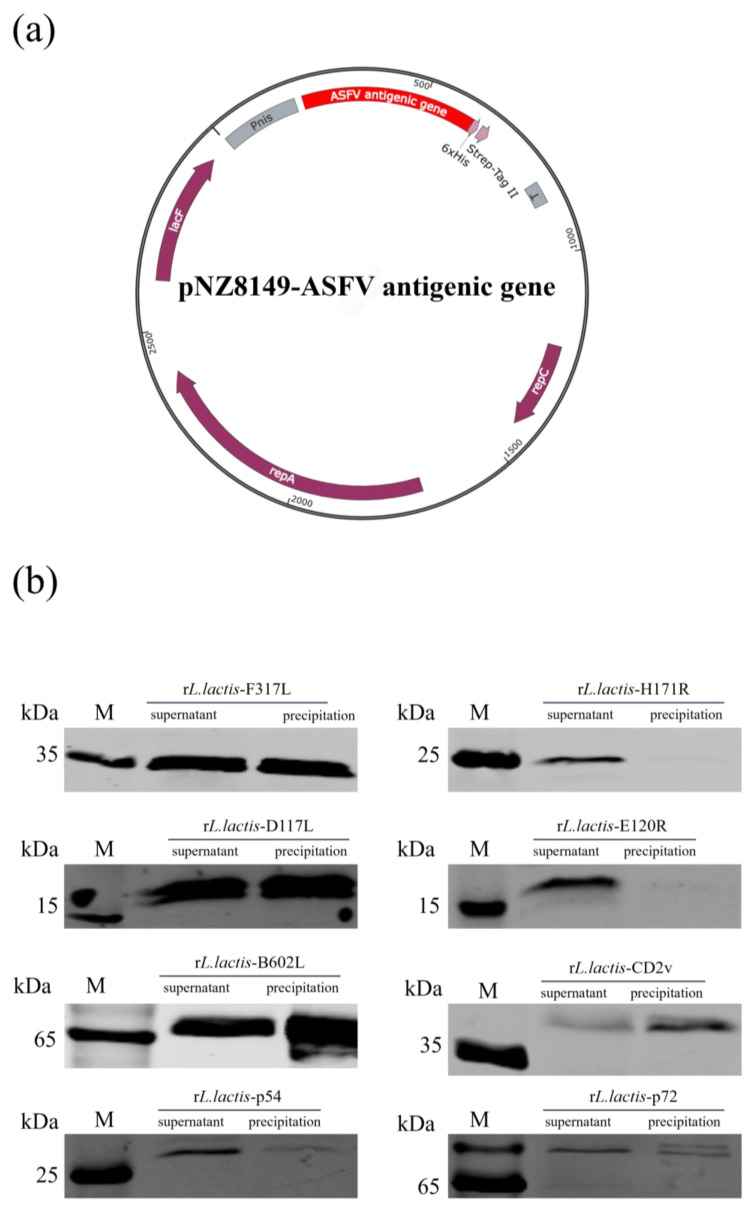
The construction of the recombinant plasmids and identification of soluble expressions of the eight ASFV antigens. (**a**) A schematic diagram of the recombinant plasmid expressing ASFV antigen. (**b**) Identification of the ASFV protein expressions of the r*L. lactis* strains by Western blotting. The r*L. lactis* strains were induced to express the proteins. Following ultrasonic disruption, the supernatant and precipitate were collected and subjected to SDS-PAGE electrophoresis to assess the expression status of the target proteins in these fractions.

The plasmid used to express the antigens of ASFV is pNZ8149, which can induce the expression of exogenous proteins in the presence of nisin. Given that the fusion of eight genes encoding ASFV antigens into a single plasmid might complicate the plasmid construction process and potentially impair the expression of the proteins, we constructed eight recombinant plasmids, each expressing only one ASFV antigen. However, the construction methods for these plasmids were largely similar. In summary, we obtained a linearized plasmid, pNZ8149, through inverse PCR and subsequently generated recombinant plasmids by performing homologous recombination between the amplified eight ASFV genes and the linearized pNZ8149, respectively (Figure 1a). To assess the expression levels and localization of the eight ASFV antigens, the r*L. lactis* strains expressing the respective ASFV antigens were induced with nisin. Subsequently, we analyzed the expression of the ASFV antigens in both the supernatant and precipitate of the ultrasonically lysed samples by Western blotting. The results demonstrate that all eight proteins, ASFV F317L (40 kDa), H171R (24 kDa), D117L (16 kDa), E120R (20 kDa), B602L (71 kDa), CD2v (43 kDa), p54 (22 kDa), and p72 (75 kDa), could be expressed in a soluble form (Figure 1b).

### 3.2. Stable Inheritance of Recombinant Plasmids Without Affecting Bacterial Strain Growth

**Figure 2:**
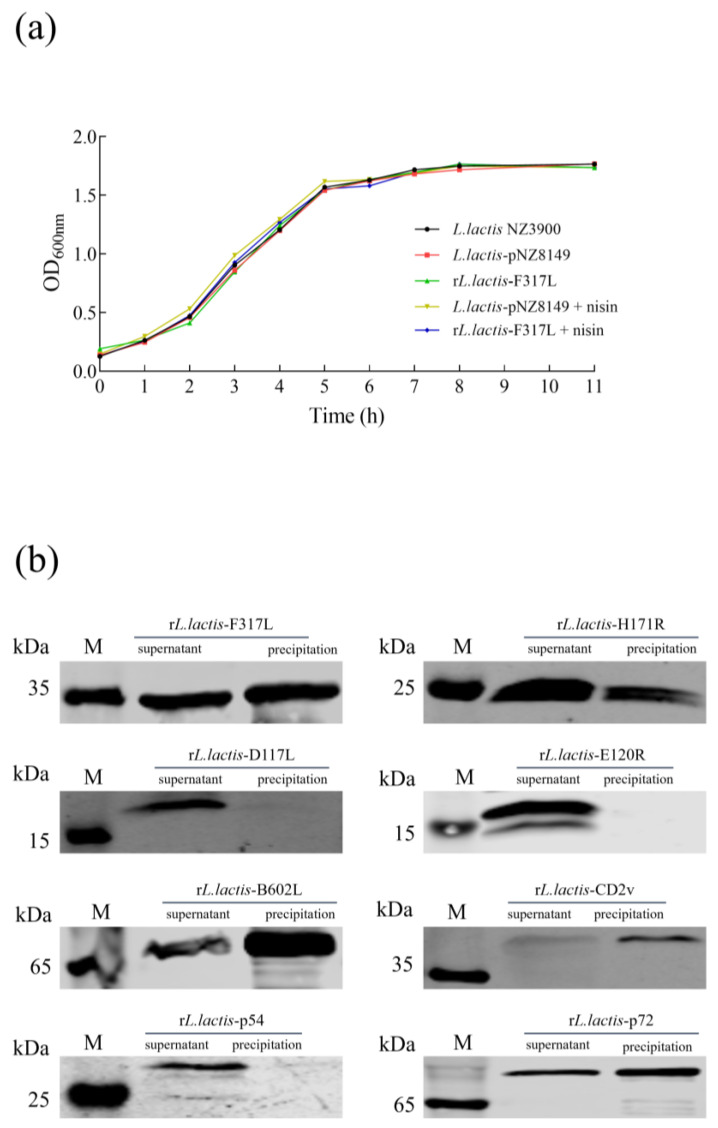
The growth characteristics and genetic stability of the r*L. lactis* strains. (**a**) The growth curve of the r*L. lactis* strains. Initially, the OD_600nm_ values of the cultures were measured. Subsequently, the OD_600nm_ values were recorded at hourly intervals until the bacterial stationary phase was reached. Utilizing these data points, the growth curves were constructed. (**b**) The detection of ASFV protein expressions in the 22nd-generation r*L. lactis* strains by Western blotting. The eight r*L. lactis* strains were subcultured daily, and upon reaching the 22nd generation, protein expressions were induced, and the supernatant and precipitate of the strain were collected using ultrasound to detect the presence of the target protein in these components.

In order to evaluate the growth characteristics of the eight constructed r*L. lactis* strains, the growth curve of r*L. lactis*-F317L was assessed as the model strain. The OD_600nm_ values of r*L. lactis*-F317L, *L. lactis*-pNZ8149, and *L. lactis* NZ3900 were measured and recorded at 1 h intervals. This monitoring was continued until the bacteria reached the stationary phase, following which growth curves were plotted based on the collected data. The results show that there was no significant difference in the growth characteristics between the recombinant strain and the parental strain (Figure 2a). To verify the genetic stability of the r*L. lactis* strains, serial passages were conducted. At the 22nd generation, the r*L. lactis* strains were induced to express the corresponding ASFV antigens, and the expressions of these proteins were confirmed by Western blotting. The results indicate that the eight r*L. lactis* strains exhibited robust genetic stability. Specifically, at the 22nd generation, none of the recombinant plasmids had been lost, and stable expression of the eight ASFV antigens (F317L, H171R, D117L, E120R, B602L, CD2v, p54, and p72) was maintained (Figure 2b).

### 3.3. Intramuscular Injection of rL. lactis-Mix Induced High-Level Serum IgG Antibodies in Immunized Mice

**Figure 3:**
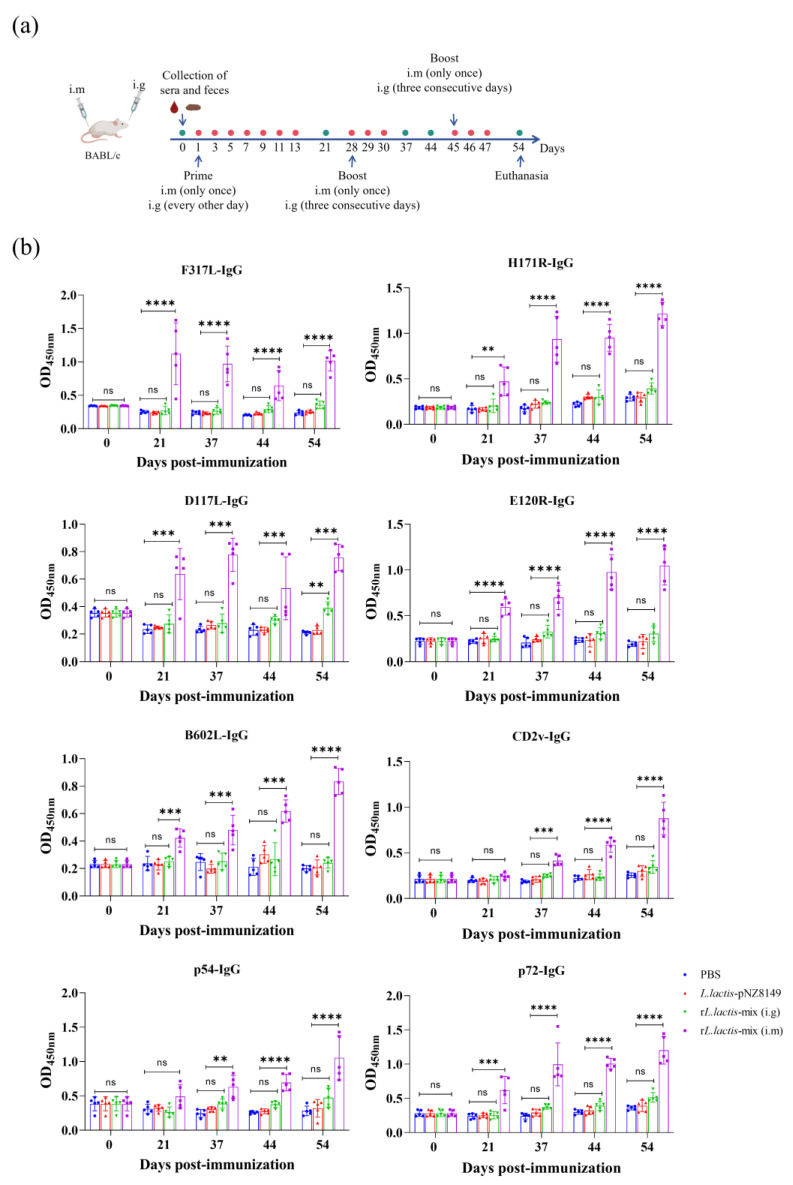
The mouse immunization schedule and sera IgG antibodies levels. (**a**) The mouse immunization schedule. The primary immunization, followed by oral gavage immunization on the subsequent day for a continuous period of 14 d. Two weeks later, a booster immunization was administered via oral gavage for three consecutive days. After another two weeks, a second booster immunization was given for three days. Each immunization was accompanied by a single intramuscular injection. (**b**) The level of specific IgG antibodies in the sera of the immunized mice. Each ASFV antigen protein was coated in 96-well microplates, and the serum specific IgG antibodies against different antigens in the immunized mice were detected by ELISA. The data were analyzed by a one-way analysis of variance. ns, *p* ≥ 0.05; **, *p* < 0.01; ***, *p* < 0.001; ****, *p* < 0.0001.

To evaluate the immunogenic efficacy of the r*L. lactis*-mix, experimental mice were immunized with the 8 × 10^9^ CFU of r*L. lactis*-mix through intramuscular injection and oral gavage. Additionally, the *L. lactis*-pNZ8149 group mice were immunized with 8 × 10^9^ CFU of *L. lactis*-pNZ8149 via oral gavage, while the PBS group mice were immunized with 200 μL of PBS administered oral gavage. Sera and fecal samples were collected from the immunized mice at various time points (Figure 3a). In this study, the eight ASFV antigens were expressed and purified by prokaryotic expression system, and an indirect ELISA detection method was established to detect the specific IgG antibodies in the sera of immunized mice. The results show that the r*L. lactis* intramuscular immunization induced high-level serum IgG antibodies 7 d post-primary immunization, which were maintained until 7 d post-second booster immunization. Unfortunately, the mice in the oral gavage group did not exhibit a significant increase in sera antibodies compared with the control group (Figure 3b).

### 3.4. Oral Gavage of rL. lactis-Mix Induced High-Level sIgA Antibodies in Immunized Mice at the Early Stage of Immunization

**Figure 4:**
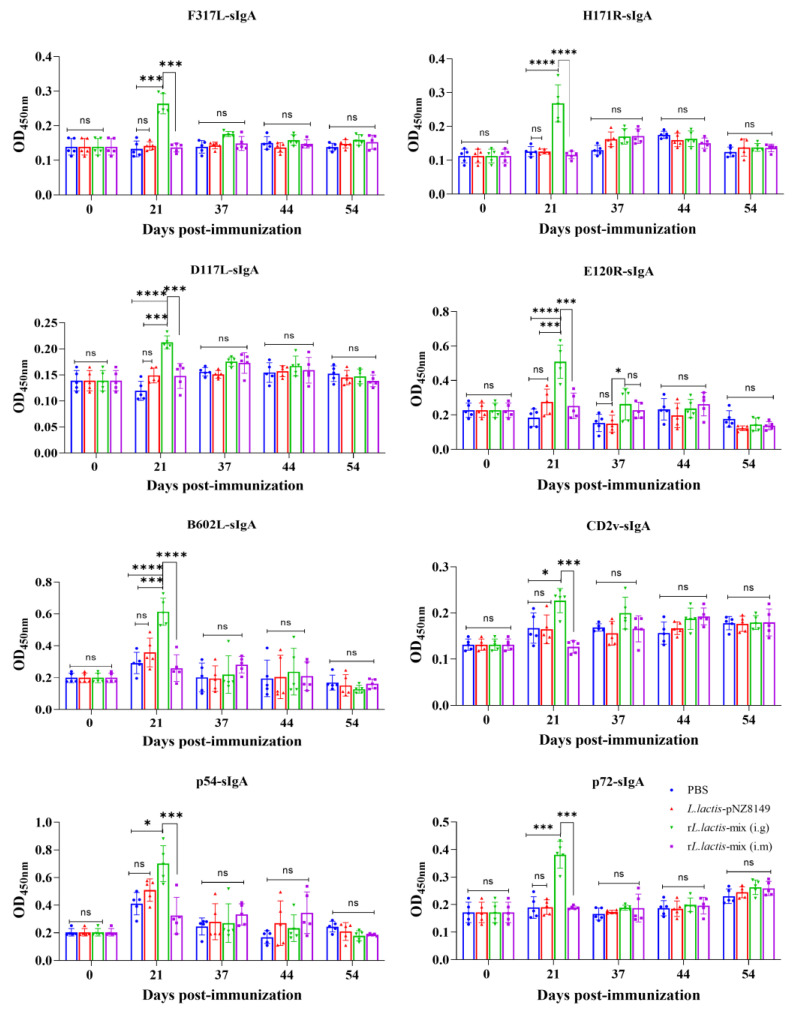
The sIgA antibodies in the feces of the immunized mice. Each ASFV antigen protein was coated in a 96-well microtiter plate, the levels of sIgA antibodies in the mice feces were detected by ELISA, and the statistical differences in the results were analyzed by a one-way analysis of variance. ns, *p* ≥ 0.05; *, *p* < 0.05; ***, *p* < 0.001; ****, *p* < 0.0001.

In order to evaluate the mucosal immune responses induced by the r*L. lactis*-mix, the feces of the immunized mice were collected at various time points, and the level of sIgA antibodies in the feces were detected by ELISA. The results show that sIgA antibodies were present in the feces of mice oral gavage with the r*L. lactis*-mix at 7 d after the primary immunization. However, in the fecal samples collected at the subsequent time points, the level of sIgA in the fecal samples of the r*L. lactis*-mix oral gavage group decreased to having no significant difference from that of the control group. Furthermore, there was no significant difference in the level of sIgA antibodies in feces between the r*L. lactis*-mix intramuscular injection group and the control group (Figure 4). Given that the intestinal mucosa provides a more precise indication of mouse intestinal sIgA levels, intestinal mucosal samples were collected from euthanized mice, and their sIgA levels were evaluated. The results show no significant difference in the level of intestinal mucosal sIgA between the mice in the r*L. lactis*-mix oral gavage group and the control group (Figure S1).

### 3.5. Intramuscular Injection of rL. lactis-Mix Elevated Sera IFN-γ and IL-10 in Immunized Mice

**Figure 5:**
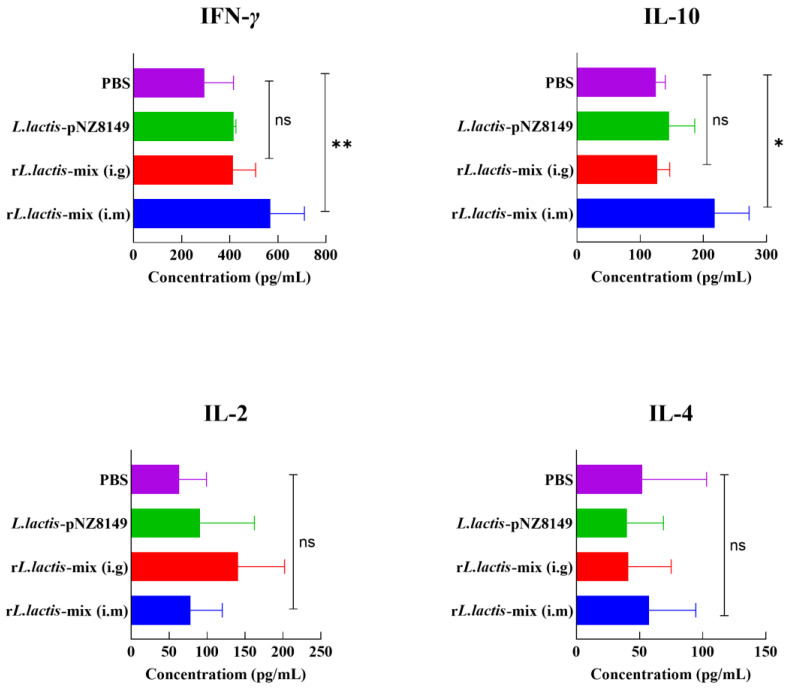
The levels of the Th1 cytokine subset (IFN-*γ*, IL-2) and the Th2 cytokine subset (IL-4, IL-10) in the sera of immunized mice. ns, *p* ≥ 0.05; *, *p* < 0.05; **, *p* < 0.01.

In order to evaluate the effect of the r*L. lactis*-mix immunization on the innate immunity of mice, we evaluated the levels of cytokines, including IL-2, IL-4, IL-10, and IFN-γ in the sera of mice. IFN-γ and IL-2 are cytokines produced by Th1 cells, which can promote the activation and proliferation of cytotoxic T lymphocytes (CTLs), macrophages, and NK cells and enhance the number of CD8^+^ T cells and innate immune responses. IL-4 and IL-10 belong to Th2 cytokine subset, which promote B cell proliferation and differentiation into plasma cells, thereby initiating antibody secretion, enhancing humoral immune response, and inhibiting inflammation. The results show that the levels of both IFN-γ and IL-10 in the sera of the mice in the r*L. lactis*-mix intramuscular injection group were significantly higher compared with those in the other groups (Figure 5).

To further evaluate the level of cellular immune response mediated by the mice immunized with the r*L. lactis*-mix, we determined the proliferation of spleen lymphocytes in the mice. Lymphocyte proliferation serves as an indicator of lymphocyte activity and functional status in response to relevant stimuli. The results show that there was no significant difference in the stimulation index of spleen lymphocytes among the four groups of mice spleen lymphocyte stimulated with 10^5^ TCID_50_ ASFV (Figure S2).

## 4. Discussion

Given the persistent threat posed by ASF to the global pig industry, the development of effective and safe vaccines has become a matter of urgency. Various strategies have been explored for developing ASF vaccines, yet each approach harbors distinct limitations [29]. ASFV naturally infects pigs primarily through contact or aerosols, breaching the mucosal barrier and leading to disease onset [30]. Currently, ASF vaccine candidates are predominantly designed for intramuscular administration, which typically fails to elicit a mucosal immune response. Consequently, recombinant ASF vaccines employing *L. lactis* may offer a promising approach to prevent ASFV invasion during the early stages of infection. In this study, we constructed eight r*L. lactis* strains expressing ASFV antigens by utilizing the NICE expression system. These eight r*L. lactis* strains included r*L. lactis*-F317L, r*L. lactis*-H171R, r*L. lactis*-D117L, r*L. lactis*-E120R, r*L. lactis*-B602L, r*L. lactis*-CD2v, r*L. lactis*-p54, and r*L. lactis*-p72. Subsequently, a mixture of these eight r*L. lactis* strains was administered to mice via both intramuscular injection and oral gavage for immunization.

The results indicate that intramuscular injection of the r*L. lactis*-mix successfully elicited high-level IgG antibodies in the sera of mice, whereas oral gavage administration of the same r*L. lactis*-mix failed to induce specific IgG antibody production in the sera. This discrepancy could be attributed to the intracellular localization of the recombinant proteins, which might hinder efficient recognition and capture of the vaccine antigens by the intestinal mucosal epithelium, thereby impeding the induction of an effective immune response. In contrast, the r*L. lactis*-mix in the intramuscular injection group was disrupted by ultrasound, making the proteins more accessible to immune cells in the bloodstream and subsequently stimulating an immune response. Consistent with our findings, previous researchers constructed the r*L. lactis*-OptiVP2-RCK, which co-expressed the primary antigen VP2 of infectious bursal disease virus and the RCK protein of an intestinal *Salmonella* strain. Despite detecting no specific IgG antibodies in the sera following oral gavage immunization, neutralizing antibodies were present and conferred protection to chickens [31]. The authors speculated that differences in protein structure, as well as distinctions between mucosal and systemic immunity, could account for these observations.

The utilization of *L. lactis* as a vaccine vector is notably advantageous due to its capacity to induce mucosal immune responses via mucosal immunization. sIgA, the predominant antibody in mucosal secretions, offers protection against pathogen adhesion and intestinal barrier penetration. In this study, we observed a significant elevation in sIgA antibody levels in the feces of the mice immunized via oral gavage with the r*L. lactis*-mix on the 7 d post-primary immunization. However, following booster immunization, the sIgA levels in the feces of these mice decreased, showing no significant difference compared with the control groups. This decline may be attributed to the early-secreted nature of sIgA, as studies suggest its primary role during the initial secretory phase of immunization [32]. In contrast to systemic immunity, the mucosal immune system not only defends against pathogenic microorganisms but also tolerates a plethora of antigens and normal flora present in food [33]. The quantity, nature of the antigen, and the frequency of immunizations are crucial factors influencing the development of immune tolerance [34]. Oral immunization with small, soluble antigen doses has been demonstrated to potentially induce a reduction in host cellular immunity and the production of immune tolerance [35,36]. Therefore, the low-level expression of the ASFV antigen protein by r*L. lactis*, combined with the prolonged immune stimulation from the primary immunization, may have led to the induction of immune tolerance in the mouse intestinal tract. This could explain why the sIgA antibody levels in the feces of the immunized mice decreased to levels indistinguishable from those of the control group during the later stages of immunization and failed to stimulate an increase in lymphocyte proliferation.

Cytokines are small molecular proteins exhibiting diverse biological activities, produced by both immune and certain non-immune cells [37]. Serving as cellular signaling molecules, they regulate immune responses, participate in the differentiation and development of immune cells, mediate inflammatory reactions, stimulate hematopoiesis, and facilitate tissue repair. Specifically, cytokines such as IL-2 and IFN-*γ*, secreted by Th1 cells, augment the activation and proliferation of CTLs, macrophages, and NK cells, thereby augmenting cytotoxicity against infected cells, enhancing phagocytic activity, and strengthening CD8^+^ T cell and innate immune responses. Conversely, IL-10 and IL-4 produced by Th2 cells promote the proliferation and differentiation of B cells into plasma cells, initiating antibody secretion and fostering humoral immune responses. Numerous studies have demonstrated the potential of utilizing *L. lactis* as a carrier to enhance both Th1 and Th2 immune responses. For instance, oral administration of pNZ8149-S1/NZ3900 expressing the S1 protein of porcine epidemic diarrhea virus (PEDV) can induce high levels of IL-4 and IFN-*γ* in the sera of immunized mice [38], and using *L. lactis* as a carrier to express the gD protein of Herpes simplex virus type 1 (HSV-1) can also significantly elevate the levels of IFN-*γ* in the serum of immunized mice [39]. Similar to previous studies, oral gavage of the r*L. lactis*-mix induced higher levels of IFN-*γ* and IL-10 production in the sera of mice.

Concurrently, it is recognized as a limitation of this study that the immunogenicity and protective efficacy of these eight r*L. lactis* strains were not assessed in pigs. However, our findings provide valuable insights into the development of ASF mucosal vaccines based on *L. lactis*. The results indicate that oral gavage immunization can elicit the production of sIgA, potentially providing early protection to pigs, whereas intramuscular immunization more effectively induces the generation of serum-specific IgG antibodies. Furthermore, more experimental animals are needed to obtain more accurate and statistically significant data. Increasing the number of immunized mice may enhance the immune data mediated by r*L. lactis*. We hypothesize that surface-displayed antigens may enhance the immunogenicity by presenting antigens on the surface of the *L. lactis*, thereby increasing the chances of antigen capture by antigen-presenting cells (APCs) and potentially amplifying the initiation of immune responses. Additionally, increasing the expression levels of ASFV antigens could further boost the immunogenic properties of the vaccines.

## 5. Conclusions

In this study, we constructed eight pools of r*L. lactis* strains, each expressing different antigens of ASFV, and evaluated the immune responses induced by these strains through oral gavage and intramuscular injection in a mouse model. The results demonstrate that the intramuscular injection group induced high levels of serum-specific IgG antibodies and significantly elevated the levels of IFN-*γ* and IL-10 in the serum. In the oral gavage immunization group, a transient increase in the sIgA antibody level was observed in fecal samples 7 d after the primary immunization. This indicates that these antigens possess good immunogenicity and that the multi-antigen cocktail vaccination is safe for mice. This study provides a reference for the development and immunization of ASF vaccines using *Lactobacillus* as a live vector.

## Figures and Tables

**Table 1:** The primers used for constructing the recombinant plasmids.

Name	Sequences (5′-3′)
F317L-F	GCACTCACCATGGTTGAAACACAAATGGAT
H171R-F	GCACTCACCATGGTTGTTTATGATCTTCTT
D117L-F	GCACTCACCATGGATACAGAAACTTCACCT
E120R-F	GCACTCACCATGGCTGATTTTAATTCACCA
B602L-F	GCACTCACCATGGCTGAATTTAATATT
CD2v-F	GCACTCACCATGATTATTCTTATTTTTCTT
p54-F	GCACTCACCATGGATTCAGAATTT
p72-F	GCACTCACCATGGCTTCAGGTGGA
Common-R	AAGCTTGAGCTCTCACTTCTCGAACTGGGG
pNZ8149-F	GGTACCACTAGTTCTAGAGAGCTC
pNZ8149-R	GCATGCCTGCAGTACCCATGGTGA

Common-R represents the same reverse primer for all the target genes.

**Table 2:** The immunization doses and routes of r*L. lactis* in mice.

Group	Amount	Immunogens	Immunizing dose	Immunization route
A	5	r*L. lactis*-mix	8 × 10^9^ CFU	oral gavage
B	5	*L. lactis*-pNZ8149	8 × 10^9^ CFU	oral gavage
C	5	PBS	200 μL	oral gavage
D	5	r*L. lactis*-mix	8 × 10^9^ CFU	intramuscular injection

## Data Availability

The dataset is available from the corresponding author upon reasonable request.

## Supplementary Materials

The following supporting information can be downloaded at: https://www.mdpi.com/article/10.3390/vetsci12020140/s1, Figure S1: The sIgA antibodies in the intestinal mucosa of the immunized mice; Figure S2: T lymphocyte proliferation of the immunized mice.
