# Supplementary material for: Evaluation of the Immunogenicity of a Pool of Recombinant Lactococcus lactis Expressing Eight Antigens of African Swine Fever Virus in a Mouse Model
Source: Vet Sci. 2025 Feb 7;12(2):140. doi: 10.3390/vetsci12020140 (PMC11861804; doi:10.3390/vetsci12020140)
Supplement: Supplementary file 1 [file vetsci-12-00140-s001.zip › vetsci-3371820-supplementary.pdf]

Supplementary Material

1 Supplementary Figures

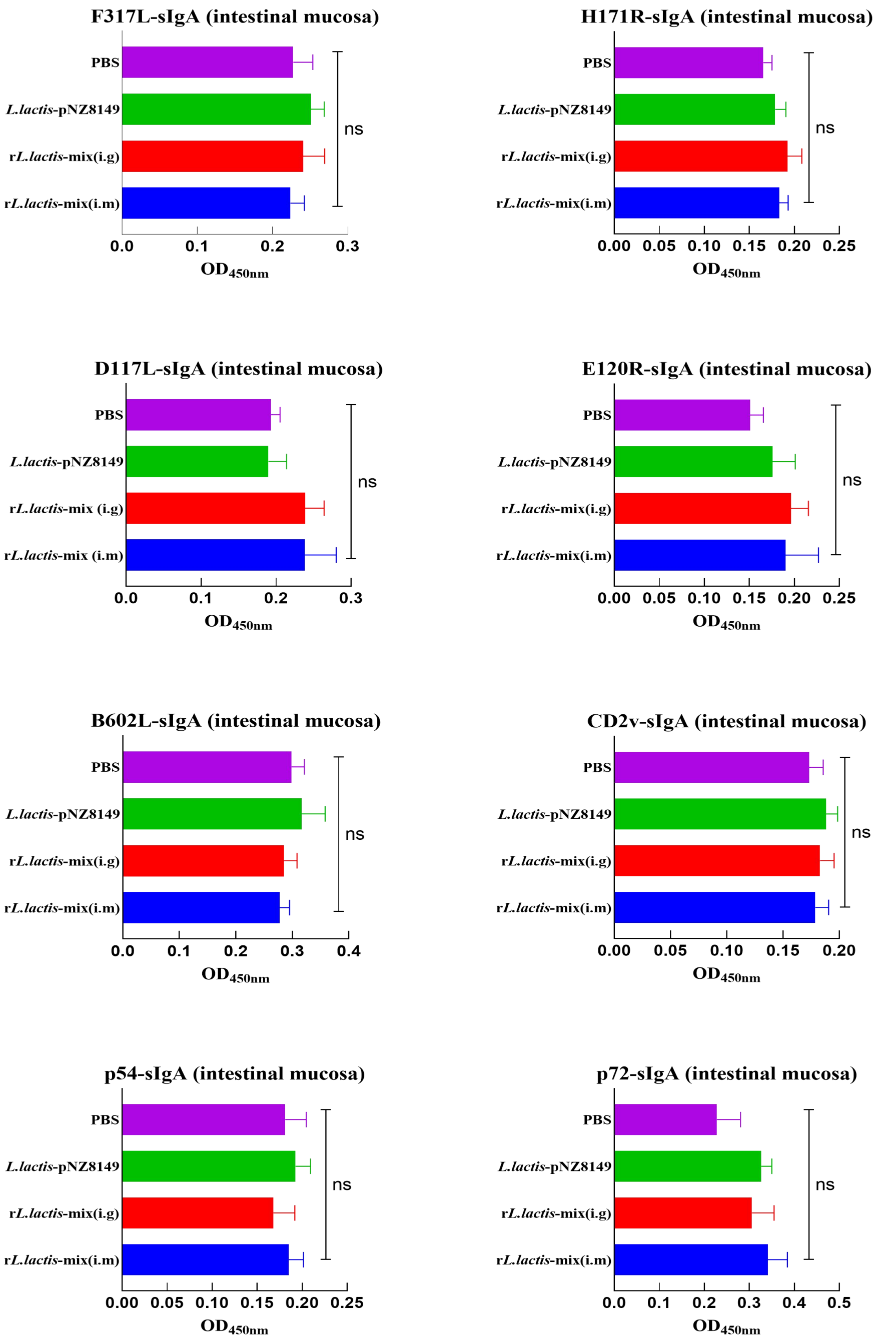

Figure S1. The sIgA antibodies in the intestinal mucosa of the immunized mice. Each ASFV antigen protein was coated in a 96-well microtiter plate, the level of sIgA antibodies in the intestinal mucosa of mice were detected by ELISA, and the difference of the results was analyzed by a one-way analysis of variance. ns, $p \geq 0.05$.

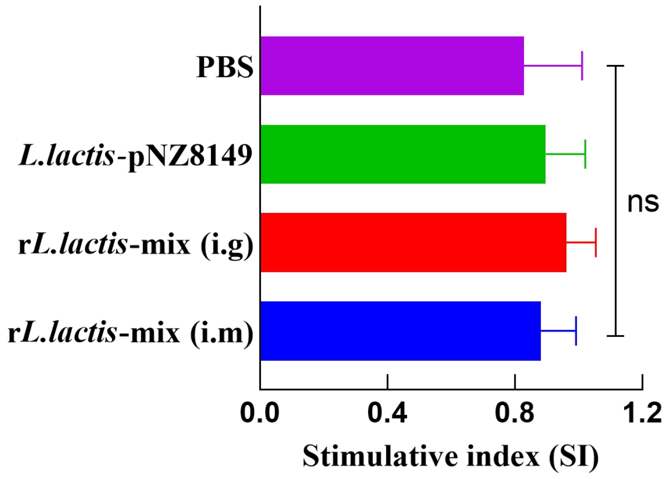

Figure S2. T lymphocyte proliferation of the immunized mice. Spleen lymphocytes were seeded into 96-well microplates at a concentration of 5×10^6 cells/ml, 50 μL per well, and stimulated with 10^5 $TCID_{50}$ ASFV as a specific antigen. The plates were cultured in a 37°C for 72 h, followed by CCK-8. After incubation at 37°C for 3 h in the dark, the absorbance at OD_{450nm} was measured using a microplate reader. ns, $p \geqslant 0.05$.
